# MicroRNA based Pan-Cancer Diagnosis and Treatment Recommendation

**DOI:** 10.1186/s12859-016-1421-y

**Published:** 2017-01-13

**Authors:** Nikhil Cheerla, Olivier Gevaert

**Affiliations:** 1 Monta Vista High School, Cupertino, CA USA; 2 Stanford Center for Biomedical Informatics Research (BMIR), Department of Medicine, and Department of Biomedical Data Science, Stanford University, 1265 Welch Rd, Stanford, CA USA

**Keywords:** miRNA, Cancer diagnosis, Pan-cancer, TCGA dataset

## Abstract

**Background:**

The current state-of-the-art in cancer diagnosis and treatment is not ideal; diagnostic tests are accurate but invasive, and treatments are “one-size fits-all” instead of being personalized. Recently, miRNA’s have garnered significant attention as cancer biomarkers, owing to their ease of access (circulating miRNA in the blood) and stability. There have been many studies showing the effectiveness of miRNA data in diagnosing specific cancer types, but few studies explore the role of miRNA in predicting treatment outcome.

**Methods:**

Here we go a step further, using tissue miRNA and clinical data across 21 cancers from the ‘The Cancer Genome Atlas’ (TCGA) database. We use machine learning techniques to create an accurate pan-cancer diagnosis system, and a prediction model for treatment outcomes. Finally, using these models, we create a web-based tool that diagnoses cancer and recommends the best treatment options.

**Results:**

We achieved 97.2% accuracy for classification using a support vector machine classifier with radial basis. The accuracies improved to 99.9–100% when climbing up the embryonic tree and classifying cancers at different stages. We define the accuracy as the ratio of the total number of instances correctly classified to the total instances. The classifier also performed well, achieving greater than 80% sensitivity for many cancer types on independent validation datasets. Many miRNAs selected by our feature selection algorithm had strong previous associations to various cancers and tumor progression.

Then, using miRNA, clinical and treatment data and encoding it in a machine-learning readable format, we built a prognosis predictor model to predict the outcome of treatment with 85% accuracy. We used this model to create a tool that recommends personalized treatment regimens.

Both the diagnosis and prognosis model, incorporating semi-supervised learning techniques to improve their accuracies with repeated use, were uploaded online for easy access.

**Conclusion:**

Our research is a step towards the final goal of diagnosing cancer and predicting treatment recommendations using non-invasive blood tests.

**Electronic supplementary material:**

The online version of this article (doi:10.1186/s12859-016-1421-y) contains supplementary material, which is available to authorized users.

## Background

MicroRNAs (also known as miRNAs) are a family of non-coding regulatory RNA genes that are involved in RNA silencing and downregulation of gene expression. They accomplish this by binding to mRNA, preventing translation (translational silencing) and speeding up deadenlyation (Poly-A tail breakdown). The first miRNA was discovered in 1993 in the roundworm *Caenorhabditis elegans* [1]. Since then, our knowledge of miRNA has grown exponentially. MiRNA can function to prevent cell division and drive terminal differentiation. An implication of this is that downregulation of some miRNAs might play a causal role in the generation or maintenance of tumors [2, 3]. Calin et al. [4] first reported miRNA’s role in cancer, after finding frequent deletions and dysregulation of two miRNAs in lymphocytic leukemia. Since then, many studies have established links between miRNA and oncogenesis. MiRNA expression profiles have previously been used to classify cancer and normal tissues and as biomarkers for prognosis [5–8].

In addition to suggesting the presence of cancer, miRNA expression also contains valuable information about the type, severity and prognosis of tumors [9]. This implies that miRNA profiles could potentially be used to recommend tumor-specific treatments instead of the “one-size-fits-all’ treatments that are often recommended by doctors.

In multiple studies, miRNA profiles of tumor tissue and adjacent normal tissue have been used to create predictive models for specific tumors. These studies focused on a relatively small number of samples. Lu et al. [6], after analyzing 217 mammalian miRNAs from 333 tumor samples consisting of tumors of lung, lymphoma and bone, discovered that miRNA expression profiles of tumors cluster based on developmental lineage. They used hierarchical clustering analysis to show that cancers of epithelial origin are clearly differentiated from cancers of the gastrointestinal tract. They further used clustering analysis among the samples of acute lymphocytic leukemia (ALL) to discover that different ALL subtypes tend to cluster together. Rosenfeld, Nitzan et al. [10] studied miRNAs from tumor and metastatic cancer to prove that they could predict the cancer tissue of origin (22 tumor types into 7 different tissue types) using KNN and decision tree classifiers. They achieved 89% classification accuracy during validation. Both these studies are limited by the number of samples (less than 400 samples) and the focus on classifying tissue of origin, where multiple tumors originating from the same tissue are classified as one class. Identifying the actual cancer type (as opposed to the tissue of origin) with as few miRNA as possible helps make it possible to diagnose specific cancer types from miRNA expression levels of circulating tumor cell miRNA in the blood [11, 12].

Although extensive research has been done on expression levels of specific miRNA to predict the prognosis of diseases like cancer, very few researchers explored the next natural topic: the role of miRNA expression profiles from the tumor tissue in predicting how successful a cancer treatment will be. Villaruz et al. [13] studied the effectiveness of miRNA as biomarkers in predicting the outcome of melanoma for patients who took a specific treatment. However, there is not enough previous pan-cancer analysis of predicting treatment effectiveness using miRNA data. With the advent of the TCGA pan-cancer analysis project, vast amounts of molecular and clinical data from tumorous and normal tissues, spanning over 34 different types of cancers, are at the fingertips of researchers [14].

In this study, we used the vast amount of tissue miRNA data available for different cancer types in the TCGA pan-cancer project to build a parsimonious multivariate model that can accurately classify cancer type. Our results show that the support vector machine based model could classify individual cancer types with an overall accuracy of 97.2% with most of the per-cancer sensitivities well above 95%, as well as classifying with above 80% sensitivity for most cancers on an independent validation set. Next, we investigated whether prognosis could be predicted given clinical information, the course of treatment taken and miRNA expression levels at the time of diagnosis. By combining miRNA expression data with clinical information in a machine learning-parseable format, we were able to predict the prognosis with 85% accuracy. Because this result was 8% more accurate than the accuracy without miRNA data, we showed that miRNA data does make a significant impact on prognosis. Finally, we built a web-based tool where a user can upload miRNA expression and clinical data and receive an automated diagnosis. We added semi-supervised learning to our web-based tool, and the tool automatically re-trains the classifiers with new uploaded data.

## Methods

### Data preprocessing and modeling

We used the R language (version 3.2.3) and CRAN package ‘caret’ version 6.0 for preprocessing and building classifier models [15]. The WEKA 3.6.13 feature suite was used for feature selection algorithms [16]. For both cancer classification and treatment recommendation, we used the normalized miRNA expression profiles from TCGA pan-cancer analysis project [17]. TCGA database has miRNA samples for 34 different tumor types obtained from GPL11154 (Illumina Hiseq 2000) platform. However, some tumor types were discarded due to low sample sizes (less than five samples per tumor type). After this selection, 5229 samples from 21 different tumor and normal tissues remained (see Table 1). These 21 cancer types span across various organs and systems. A few of these cancer types (Lung Squamous Cell Carcinoma, Stomach Adenocarcinoma, Liver Hepatocellular Carcinoma, Thyroid Carcinoma, Kidney Chromophobe and Kidney Renal Papillary Cell carcinoma) have significant number of normal tissue samples as well in the dataset. But, many cancer types either have very few corresponding normal samples or no normal samples. Also, the samples have missing miRNA values. These missing miRNA value estimation was done using a k-Nearest-Neighbor imputation algorithm [18]. A threshold value of 0.2 was used to eliminate miRNA features that were not present in more than 20% of samples. Finally, the miRNA data was log transformed and standardized to zero mean and unit variance.

**Table 1:** Distribution of the cancer and normal samples in the dataset used to build the predictive model (TCGA cancer classifier)

Organ/System	Cancer	Cancer acronym	Normal samples	Cancer samples
Thymus	Thymoma	THYM	2	124
Lung	Lung Squamous Cell Carcinoma	LUSC	45	342
Pancreas	Pancreatic Adenocarcinoma	PAAD	4	179
GI tract	Cholangiocarcinoma	CHOL	9	36
	Esophageal Carcinoma	ESCA	13	185
	Stomach Adenocarcinoma	STAD	41	395
Liver	Liver Hepatocellular Carcinoma	LIHC	50	374
Thyroid	Thyroid Carcinoma	THCA	59	510
Adipose	Adrenocortical carcinoma	ACC	0	80
Lymph	Diffuse Large B-cell Lymphoma	DLBC	0	47
Heart	Mesothelioma	MESO	0	87
Reproductive	Cervical Squamous Cell and Endocervical Adenocarcinoma	CESC	3	309
	Ovarian Serous Cystadenocarcinoma	OV	0	461
	Testicular Germ Cell Tumors	TGCT	0	156
Urinary	Uterine Carcinosarcoma	UCS	0	56
Kidney	Kidney Chromophobe	KICH	25	66
	Kidney Renal Papillary cell carcinoma	KIRP	34	292
Brain	Brain Lower Grade Glioma	LGG	0	526
Peripheral Nervous System	Pheochromocytoma and Paraganglioma	PCPG	3	184
Epidermis	Skin Cutaneous Melanoma	SKCM	2	450
	Uveal Melanoma	UVM	0	80

Note that not all the cancer types have normal samples. Even though the TCGA dataset has about 33 cancer types, many cancer types were removed due to lack of data (less than 5 samples) and in the end 21 cancer types as listed in this table were used for the classification

### Building a cancer classifier

We built several multi-class classifiers (SVM linear, SVM radial, Random Forest, Linear Discriminant Analysis and Naïve Bayes) and validated them using 10-fold cross validation, repeated ten times. These classifiers are trained to classify 21 different cancer types and the normal tissue type from the miRNA expression values. During the training (for each fold), we used two-stage feature selection algorithm. In the first stage, we used a correlation based feature selection algorithm [19] to remove features that showed no meaningful Pearson correlation with the output. During the second stage, recursive feature elimination algorithm (from the caret package in R) was used to narrow down the feature set to 60 features while picking a strong feature set that would not compromise the classification performance.

The classifier with highest overall accuracy, aggregated across all validation tests, was selected and analyzed for misclassifications. Overall accuracy (or the classifier accuracy) is defined the ratio of all correctly classified instances over the total instances during the testing phase of each iteration in the 10-fold cross validation. Similarly, per-class accuracy is defined as the ratio of the correctly classified instances of a particular class to the total number of instances of that class. Next, we used the kappa statistic -- as a measure of the agreement as compared to the likely agreement due to chance alone -- to evaluate the classifiers. We also measured per class training sensitivities and specificities of the classifier that had the highest overall accuracy and kappa statistic.

Since the feature selection is done during training phase of each of the ten iterations in 10-fold cross validation, features selected could differ between iterations. We took the common features from all the iterations and analyzed the features using the IPA core analysis tool (IPA ® QIAGEN, Redwood City, www.qiagen.com/ingenuity) to find the diseases/functions and networks enriched by this subset. This analysis gives insight into whether our classifier performance is driven by tissue specific miRNAs or cancer/tumor specific miRNAs.

### Tissue/Organ/Origin prediction

Studies have shown that miRNA from tissues with similar anatomical locations correlate well [20]. Thus, we further investigated whether miRNA expression values from cancerous tissues also correlate along anatomical locations and if the correlation follows tissue development hierarchies. To formalize this, each cancer site was assigned to leaf nodes in the embryonic development tree [21] (Fig. 2). Then, by walking up the tree, classification was compared at three stages of the developmental tree. For example, stomach adenocarcinoma was classified as a cancer of GI tract at stage 3, a cancer of gut tube origin at stage 2 and a cancer of endoderm origin at stage 1.

### Independent validation

Next, for independent validation of our SVM based classifiers, we obtained three miRNA datasets from GEO database [22] and validated the performance using those datasets. Due to the lack of open access datasets for the majority of cancer types in our 21-cancer SVM classifier, we had to use cancer types that our model was not trained on. We used our Stage III classifier, which classifies these cancers by tissue/organ type, with these datasets. The GSE2564 series uploaded by Jun et al. [6] contains miRNA expression values for 200+ cases spanning across 12 different cancer types. The GSE68839 series uploaded by Vergani et al. [23] contains miRNA data for eight melanoma samples. The GSE21847 series uploaded by Montes-Moreno et al. [24] had 29 samples of B-cell Lymphoma. We used log2 normalized expression values from these data sets and further standardized the datasets individually to have zero mean and unit variance.

### Prognosis prediction

The TCGA database contains clinical data for some, but not all, patients. This data includes clinical data such as ethnicity, gender, the type of treatments and drugs used, and a description of the outcome (e.g. whether the cancer recurred or not). Out of the 5229 patients, complete clinical information is available for about 710 patients. When selecting the samples for building the treatment recommendation tool, only the samples with the below criteria were retained: (1) contains clinical information: gender, age and ethnicity, the type of treatment received and if the tumor went under remission after 300 days or more. (2) There are at least five samples for each cancer type (3) There are at least five samples for each unique treatment.

This data set is further divided into training and testing with 75–25 split. All the unique treatments from the training samples were extracted. In order to represent the treatment in a form that machine learning models could easily interpret, treatment data items were first converted into a canonical bag-of-strings form of “general treatment, specific method;” and then sorted in alphabetical order and combined (Additional file 1). First, we used the Levenshtein/edit distance to define a similarity metric between patients with different treatments. Next, we mapped these distances into 3-dimensional space. A 3-dimensional point P_k_ was attached to each treatment k, and an attempt was made to minimize the difference between 3D distances and the “true” (Levenshtein) distance through an optimization formula: $min\;\left(\sum_{i\ne j}\left( ldist\left(i,\kern0.24em j\right) - dist\left({P}_i,\;{P}_j\right)\right)\right)$. R provides a solution to problems of this form (often called Cox scaling problems) with the package “cmdscale”. This analysis was done on the unique treatment list to obtain three numeric coordinates for each treatment. Then, a support vector machine model was trained on a combination data set featuring miRNA data, clinical data, cancer type, and treatment type (in 3D coordinate form) to predict the clinical outcome (i.e. recurrence or remission status after at least 300 days). The SMOTE algorithm [25] was used to correct class imbalance caused by low number of samples where disease reoccurred compared to remission samples. The model with highest accuracy and per-class sensitivities on the test data set was chosen as the prognosis predictor tool. The prognosis predictor model output is a score (between 0 and 1) that indicates the probability of remission.

### Web tool development

Next, we developed a treatment recommendation algorithm based on our prognosis model. The algorithm works as follows:

Given miRNA information and clinical data for a patient, this algorithm cycles through all possible treatments from the unique treatment lists obtained in the previous analysis, and for each treatment, predicts the probability of remission using the prognosis predictor tool. Then, the treatment that results in the highest probability of remission is picked as the recommended treatment.

Then, using the RStudio shiny package (version 0.12) [26], a web application was built. In this web application, our SVM classifier and the treatment recommendation tools were hosted on a server and the web application runs in a browser. Using the user interface for this application, user can upload clinical information using dialog boxes, with miRNA information in a CSV file.

The data will be sent to the servers where the application pre-processes the data, including imputing missing miRNA values (removing miRNAs with more than 20% missing), then uses the saved classification and treatment recommendation models to diagnose cancer type and makes three different treatment recommendations. A semi-supervised learning algorithm with the potential to improve the accuracy of both models was implemented. In this algorithm, the newly uploaded training samples classified with a probability over a threshold *p* = 0.95 are periodically added to the database, and the models have the opportunity to retrain and adjust based on these samples.

## Results and discussion

### Cancer diagnosis and classification

**Fig. 1:**
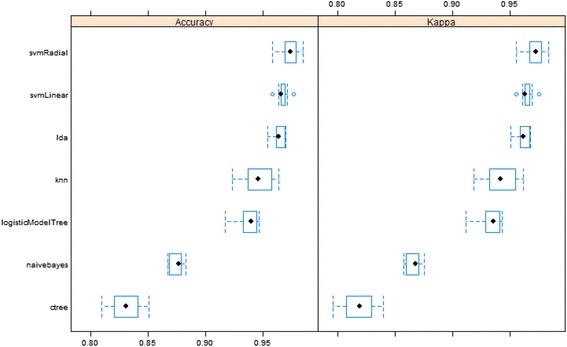
Performance metrics for multiclass classifiers. Accuracy and kappa statistics for the 7 multiclass classifiers that we evaluated, using boxplots to reflect classifier variability over multiple runs

Out of the 2588 miRNA features, 2118 miRNA were removed during pre-processing as more than 20% of the samples had missing values for these miRNA leaving a feature set of 470 miRNAs. Most of the missing miRNA values were systemically missing across all cancer types. Using this feature set, different classifiers (Naïve Bayes, Logistic Model Tree, KNN and Linear SVM) were built and the overall accuracy and kappa statistics were compared (Fig. 1).

**Fig. 2:**
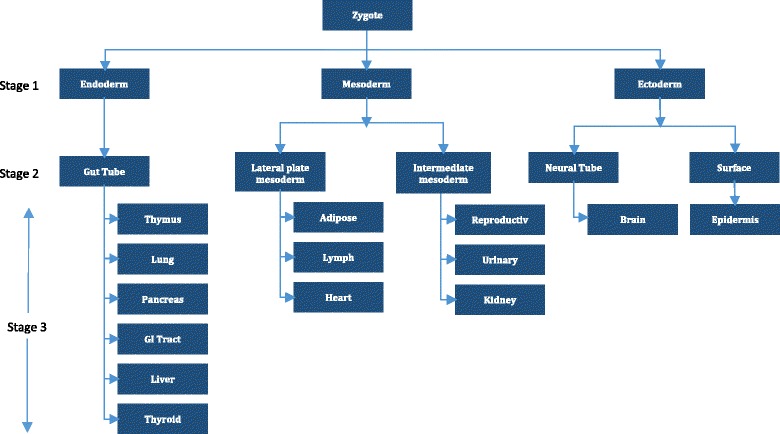
Mammalian developmental tree. Each of the 21 cancer types was assigned to its appropriate leaf node of the tree

The radial SVM classifier was chosen as it reported the highest accuracy, 97.2%. This classifier also has the highest Kappa statistic of 0.97. Per-cancer sensitivities and specificities of this classifier were listed in Table 2. Table 3 contains the confusion matrix of this classifier. Most of the sensitivities are above 90%, except for ESCA, esophageal adenocarcinoma and CHOL, cholangiocarcinoma. Further inspection of the confusion matrix in Table 3 revealed that, 11% of CHOL cases were classified as pancreatic adenocarcinoma (PAAD). 12% of the ESCA cases were classified as Stomach Adenocarcinoma (STAD) and 6% of the ESCA cases were classified as liver hepatocellular carcinoma (LIHC). The STAD, CHOL and ESCA cancers are from the gastrointestinal (GI) tract and share a common developmental origin (Fig. 2). Similarly, PAAD or cancers in the pancreas and LIHC are related to the other three cancers due to a similar developmental parent (i.e. gut tube).

**Table 2:**
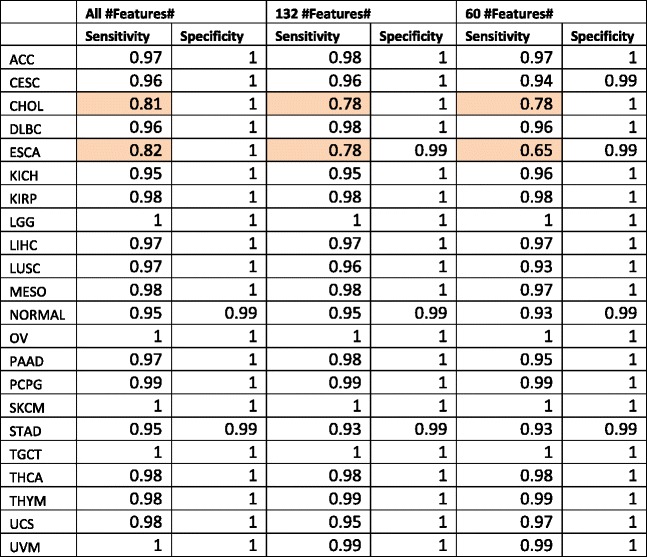
SVM classifier performance

Per-cancer performance metrics for the SVM classifier with all features and with various feature subsets selected by two-stage feature selection algorithms. The cells shaded in pink are the cancer types with sensitivities below 90%

**Table 3:**
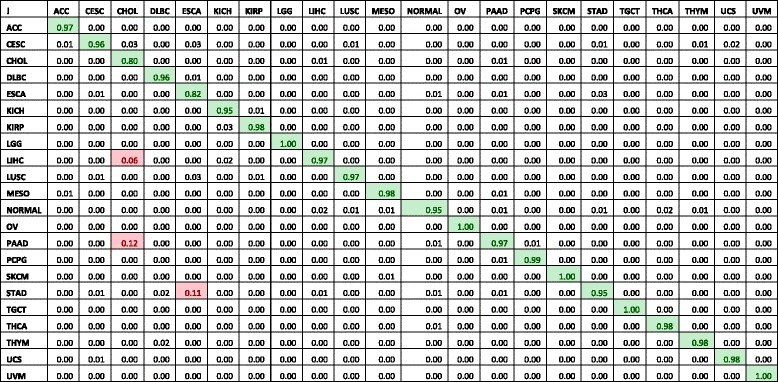
Confusion matrix for the SVM classifier

This matrix is obtained by aggregating the results of 10-fold cross validation repeated 10 times. The rows represent the predictions and the columns represent the true values. The entry values contain the fraction of the overall samples of a cancer type (represented by the column) that are predicted as the cancer type represented by the row. Cells shaded in orange-red colors represent misclassifications greater than 5% of the total samples for that cancer type. For example, for the ESCA cancer type, 11% of the ESCA cancer type samples were misclassified as STAD

Predictably, the results improve dramatically when walking up the embryonic origin tree diagram and classifying at different stages. Table 4 lists the overall sensitivities when classifying at different stages of the embryonic development tree. Sensitivities slowly increase at the higher levels with accuracies reaching to 100% at the germ layer (Stage 1). For example, the ESCA (esophageal cancer) class is detected with 82% sensitivity initially, but when it is combined with other gastrointestinal cancers to form the subtype GI tract, the sensitivity jumps to 0.98 with Stage III classifier. Then, when classified as a gut tube cancer, the sensitivity increases even further with the Stage II classifier. These increases are more than would be expected by chance aggregation alone and hint at a relation between cancer differentiation and the embryonic origin tree diagram.

**Table 4:** SVM classifier performance at different stages of the embryonic development tree

Stage I classifier			Stage II classifier			Stage III classifier		
Subtype	Sensitivity	Specificity	Subtype	Sensitivity	Specificity	Subtype	Sensitivity	Specificity
Endoderm	0.99	1	GI Tube	0.99	0.99	Thymus	0.98	1
						Lung	0.98	1
						Pancreas	0.96	1
						GI Tract	0.98	1
						Liver	0.98	1
						Thyroid	1	1
Mesoderm	0.99	1	Lateral Plate Mesoderm	0.97	1	Adipose	0.98	1
						Lymph	0.96	1
						Heart	0.98	1
			Intermediate Mesoderm	0.99	1	Reproductive	0.99	1
						Urinary	0.98	1
						Kidney	0.99	1
Ectoderm	1	1	Neural Ectoderm/Neural Tube	1	1	Brain	1	1
			Neural Crest	0.99	1	Peripheral Nervous System	0.99	1
			Surface Ectoderm	1	1	Epidermis	1	1

Climbing up the embryonic development tree, SVM classifiers were built at each stage to classify the cancers at different granularities. Stage 4 identifies the actual cancer. Stage III classifier can classify the cancers at the tissue/organ level. At Stage I, the cancer is classified as belonging to one of the germ layers

The first level of feature selection yielded 132 predictive miRNA features. The second level yielded 60 miRNA features. Table 2 also lists the per-class performance of the classifier for 132 and 60 features. The classifier built with 60 features achieved an overall accuracy of 95.5% with per-class sensitivities above 95% for many cancer types. Note that, due to the scarcity of the normal samples, all the normal tissue samples were put together under a single class in these classifiers. The per-class sensitivities and specificities of the ‘Normal” class were 95 and 99% respectively, indicating the ability of the classifier to distinguish between the many cancer types from the normal tissues.

These results suggest that miRNA can be used to predict diagnosis with extremely high accuracy, sensitivity, and specificity.

The results from IPA core analysis on the 60 miRNA features used by our classifier are summarized in Additional file 2. As seen from this table, 49 out of the 60 molecules are highly correlated with cancer. The top two disease categories enriched by our miRNA features are *metastatic solid tumor* (*p*-value of 3.27E-53) and *advanced malignant solid tumor* (*p*-value of 1.61E-50). Similarly, the top three networks enriched by these set of miRNA are related to cancer, organismal injury and abnormalities and gastro-intestinal diseases (Additional file 3: Table S1). IPA computes the *p*-value for a function/process as the measure of likelihood that the association between that function and set of molecules (in our case, miRNAs) is due to a random chance. The smaller the *p*-value (or the higher the negative log of the *p*-value), the higher the likelihood that there is a significant association. *P*-value was obtained using Benjamini-Hochberg method to account for multiple testing [27].

The high negative log p-values for association between the cancer/tumor progression and the miRNA features used by our classifier suggest that the classifier was able to select and classify these 21 different cancer types using key miRNA features previously known to be associated with many cancer types. The features were predominantly cancer specific (rather than tissue specific), hinting that tumor progression mechanisms differ at the miRNA level between tissues.

We then analyzed the top three miRNAs (ranked by our feature selection algorithm) from our 60 miRNA features. The first one, let-7 family of miRNAs (hsa-let-7i-5p, hsa-let-7d-3p) has been mentioned extensively in the literature as the miRNA precursor that regulates cell cycle progression and cancer [28–30]. Decreased expression of let-7 was known to cause unregulated cell division and tumor formation [31]. The second feature, mir-1-3p (mir-1 family) was studied by Hu et al. [32]; it has been suggested that this miRNA plays a critical role as a tumor activator for human liver hepatocellular carcinoma. Decreased expression of mir-1 could decrease proliferation and induce apoptosis. Mir-10 family (mir-10a and mir-10b) were studied extensively before for their casual role in several cancer types. Lund et al., summarized the previous studies and reported that mir-10 was dis-regulated in several cancer types including Hepatocellular carcinomas, Pancreatic cancer, B-cell Chronic Lymphocytic Leukemia and Melanoma [33]. These are all cancer types our classifier can distinguish. This IPA analysis combined with our literature search indicate that our SVM classifier was able to achieve better performance using miRNAs previously known to cause and promote tumor proliferation.

### Independent validation

The GSE2564 and GSE21847 datasets have 50 and 39 miRNA features in common with the 60 miRNA features used by our classifiers. The remaining miRNAs were imputed using the k-Nearest-Neighbor imputation algorithm [18]. GSE68839 samples had miRNA values for all the 60 miRNA features. These three datasets were normalized independently to have zero mean and unit variance.

Many cancer types in these three dataset don’t exactly match the cancers our 21-cancer type SVM classifier was trained to classify (Additional file 3: Table S2). Only Pancreatic Adenocarcinoma (PAAD) and Diffuse Large B-cell Lymphoma (DLBC) types matched one of the cancer types our SVM classifier was trained to predict – the rest were not cancer types from our dataset. To circumvent this problem, we relied on the SVM classifier at stage III (of the embryonic development tree, Table 4), to classify based on the organs and tissue of origin and predicted the cancer tissue/organ for the various cancer types in the validation set. Confusion matrix from this classification and the per class sensitivities and specificities were given in the Additional file 3 Tables S3 and S4). The cancers of epidermis, kidney, lymph nodes, pancreas reproduction systems achieved sensitivities greater than 70% (all cancers achieved high specificities). Cancers of urinary tract performed poorly with a sensitivity of 0.43 (four out of seven samples were incorrectly classified). The drop in per class accuracy, compared to our trained stage III SVM classifier could be attributed to the differences in the platforms, missing miRNA features (15% features missing in GSE2564 series and 33% missing in GSE21847 series), lower overall sample size and the fact that many of these cancers were from cancer types not in the TCGA dataset. But, the results are still extremely promising, especially considering that they were mainly on completely unseen cancer types. They suggest that our Stage III classifier was able to perform very well on an independent, unseen dataset in the presence of platform variation.

### Prognosis prediction

**Fig. 3:**
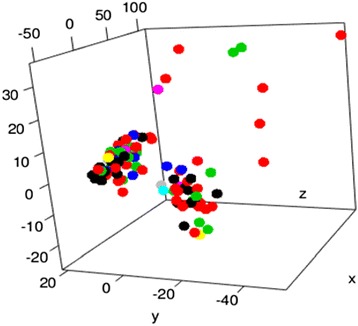
Cox scaling map. A graphical representation of the Cox scaling map of treatment space. Uses the MATLAB “jet” colormap, with *black* and *red colors* representing more prevalent treatments and *green* and *blue colors* representing less prevalent treatments. The (*x*, *y*, *z*) axes simply represent the 3 Cox-scaled coordinates assigned to each treatment. Treatments with edit distances less than 25 were further merged to form 29 unique treatments

After merging treatments with edit distance less than 25 to reduce the number of unique treatments, a cohort of 476 patients with 9 unique cancer types and 29 unique treatment types remained (Table 5) [see Fig. 3 for a graphical representation of the Cox scaling results. Additional file 1 contains a subset of the unique treatments.]. Using the clinical and miRNA data, a radial SVM classifier was trained and validated with repeated 10-fold cross validation. Table 6 lists the classifier results (aggregated over repeated cross validations) with and without the miRNA data. The prognosis predictor achieves 85% accuracy overall, showing approximately 8% increase in the accuracy after adding miRNA features to the classifier. These results suggest that miRNA play a significant role in the prognosis of cancers.

**Table 5:** Distribution of the samples used in prognosis prediction and treatment recommendation models

Cancer type	# of patients	# of unique treatments per cancer	# reoccurrence cases
CESC	52	4	9
ESCA	23	4	5
LGG	137	6	88
LUSC	15	7	3
OV	111	9	23
PAAD	45	4	32
STAD	37	5	13
TGCT	51	4	6
UCS	13	2	3

Preprocessing the cohort of 710 patients with full clinical and treatment information yielded a smaller subset of 476 patients

**Table 6:** Prognosis predictor classifier performance

	SVM with miRNA features	SVM without miRNA features
Disease sensitivity	0.86	0.76
Disease specificity	0.84	0.77
Accuracy	0.85	0.77
Kappa statistic	0.71	0.53
Accuracy standard deviation	0.03	0.05
Kappa statistic standard deviation	0.05	0.09
Tuning parameters (Sigma)	0.01	0.16

### Web tool development

The web tool was built according to specification and is located at http://www.mirnanalyze.com. The tool was tested extensively (on the web) using examples from TCGA to ensure its results matched the results obtained by standalone R scripts. As more researchers and labs begin to use the web tool developed, it will collect more unlabeled data and we will ideally be able to evaluate the effects of our semi-supervised learning algorithm.

In summary, for cancer diagnosis, we saw hints that cancers were differentiated somewhat based on embryonic tissue-of-origin. For future research, it would be interesting to explore exactly how similar cancers with the same embryonic tissue of origin are, as well as what causal factors underlie the relation between these two phenomena.

For treatment prediction, an issue affecting our accuracy negatively was the existence of “invisible” causal factors, other conditions not collected by the TCGA. The accuracies were also affected by the very few samples with full clinical and treatment data. Due to these limitations, the tool, in its current state, is not ready to be used in clinical settings. The tool would need to be trained with more features and more samples with and without remission for each treatment and cancer type.

Recently, cell free miRNAs have garnered lot of attention as potential blood based biomarker for cancer. We could potentially test our classifier performance in detecting cancers from the cell-free miRNA expression values from blood serum/plasma; this would be of immense clinical significance.

Medical automation is one of the most fruitful fields in research today. All of the work presented here and all of the future research planned are dedicated towards one specific goal; that in the near future, physicians will be able to take a blood sample from any patient suspected of having cancer, immediately analyze it online, and receive a diagnosis with multiple treatment options. The authors hope that this research is one step on the journey to that final goal.

## Conclusions

We successfully built a miRNA based classifier that can classify between 21 different cancer types with 97.2% overall classification accuracy (with per class accuracies well above 905). This performance is 7% better than the state-of-the-art. Our prognosis prediction model was able to achieve 85% accuracy in predicting the reoccurrence for a patient. We were able to use this prognosis prediction classifier to build a novel treatment recommendation system that is uploaded at www.mirnanalyze.com.

The SVM classifier for cancer diagnosis achieved an accuracy of 97.2% in repeated 10-fold cross validation tests. It is the first ever model to integrate a large cohort of patients (5229 patients) to classify 21 different types of cancers. This high accuracy was obtained due to the large dataset, careful pre-processing and imputation of the data, picking the best model and tuning classifier parameters. Many of the misclassifications made by this classifier were between cancers with similar embryonic origins, and the accuracies improved to 99.9–100% when tree-climbing up the embryonic developmental model, suggesting that cancers originating from similar stem cells have similar molecular characteristics. The two-step feature reduction yielded 60 miRNAs and a classifier using these 60 miRNA was able to achieve 95.5% overall accuracy.

A detailed functional analysis of these 60 miRNA features using the IPA tool revealed that 49 of them are highly associated with cancer and tumor metastasis (*p*-value < 0.05), suggesting that the classifier was able to pick tumor specific miRNA (as opposed to tissue specific miRNA) for these 21-cancer type classifications.

We had promising results on an independent validation dataset. Despite our dataset being composed mainly of cancer types that our SVM model was not trained on, our classifier achieved high specificities on all cancer types and around 70-80% sensitivity on all but 3. Our model’s ability to classify completely unseen cancer types from an unseen dataset indicates its generalizability.

Using clinical info, miRNA and treatment data, a prognosis predictor tool was then built that achieved 85% accuracy—an 8% improvement over a classifier that predicts prognosis only based only on clinical and treatment data. Table 6 compares AUC for the prognosis prediction with and without the miRNA data. AUC is significantly higher (0. 87) for prognosis prediction with miRNA data. Because many hard-to-measure factors such as diet, exercise, clinical history, and other clinical information that was not collected affect prognosis, such accuracy is high for a prognosis model.

Our web tool was deployed at www.mirnanalyze.com and is available for predicting cancer type as well as recommending 3 personalized treatment regimens to improve remission probability.

This research distinguishes itself through:

- First integrated 21-cancer classifier that can detect the type and presence of cancer with 97.2% overall accuracy (over 7% more accurate than all previous research).
- First attempt to model prognosis prediction given the miRNA, clinical and treatment information, and build a personalized treatment regimen recommendation tool based on miRNA profile data.
- First attempt to crowd-source diagnosis and treatment tools to the public.

## Additional files

Additional file 1: This is a word document that contains a subset of unique treatments generated by the cox scaling method. (DOCX 14 kb)
Additional file 2: This excel file contains the results from the core analysis of the 60 miRNA features. (XLSX 10 kb)
Additional file 3: This is a word document that contains supplemental tables. (DOCX 45 kb)

## Abbreviations

MiRNA: MicroRNA
TCGA: The Cancer Genome Atlas
