# Supplementary material for: MicroRNA based Pan-Cancer Diagnosis and Treatment Recommendation
Source: BMC Bioinformatics. 2017 Jan 13;18:32. doi: 10.1186/s12859-016-1421-y (PMC5237282; doi:10.1186/s12859-016-1421-y)
Supplement: Additional file 1: — This is a word document that contains a subset of unique treatments generated by the cox scaling method. (DOCX 14 kb) [file 12859_2016_1421_MOESM1_ESM.docx]

List of the top 20 treatments used in the prognosis prediction model.

[1] "chemotherapy, cisplatin; radiationtherapy, external; radiationtherapy, internal"

[2] "chemotherapy, cisplatin; radiationtherapy, external"

[3] "chemotherapy, carboplatin; chemotherapy, taxol"

[4] "chemotherapy, carboplatin; chemotherapy, paclitaxel; radiationtherapy, external"

[5] "chemotherapy, fluorouracil; radiationtherapy, external"

[6] "chemotherapy, capecitabine; radiationtherapy, external"

[7] "chemotherapy, capecitabine; chemotherapy, cisplatin; radiationtherapy, external"

[8] "chemotherapy, temodar; radiationtherapy, externalbeam"

[9] "chemotherapy, temodar"

[10] "chemotherapy, temodar; radiationtherapy, external"

[11] "chemotherapy, temozolomide; radiationtherapy, external"

[12] "chemotherapy, temozolomide; radiationtherapy, externalbeam"

[13] "chemotherapy, temozolomide"

[14] "chemotherapy, carboplatin"

[15] "chemotherapy, carboplatin; chemotherapy, paclitaxel"

[16] "chemotherapy, carboplatin; chemotherapy, taxotere"

[17] "chemotherapy, cisplatin; chemotherapy, etoposide"

[18] "chemotherapy, carboplatin; chemotherapy, taxol; chemotherapy, taxotere"

[19] "chemotherapy, carboplatin; chemotherapy, cisplatin; chemotherapy, paclitaxel"

[20] "chemotherapy, cisplatin; chemotherapy, paclitaxel"
