# Supplementary material for: MicroRNA based Pan-Cancer Diagnosis and Treatment Recommendation
Source: BMC Bioinformatics. 2017 Jan 13;18:32. doi: 10.1186/s12859-016-1421-y (PMC5237282; doi:10.1186/s12859-016-1421-y)
Supplement: Additional file 3: — This is a word document that contains supplemental tables. (DOCX 45 kb) [file 12859_2016_1421_MOESM3_ESM.docx]

**Supplementary Table 1:** IPA Core Analysis Results.

| **Name** | **p-value Range** | **Number of Molecules** |
| --- | --- | --- |
| **Diseases and Disorders** |  |  |
| Caner | 3.69E-02 - 3.27E-53 | 50 |
| Organismal Injury and Abnormalities | 3.69E-02 - 3.27E-53 | 49 |
| Reproductive System Disease | 3.69E-02 - 1.43E-47 | 42 |
| Gastrointestinal Disease | 2.97E-02 - 5.00E-45 | 42 |
| Developmental Disorder | 2.97E-02 - 2.83E-37 | 30 |
|  |  |  |
|  |  |  |
| **Molecular and Cellular Functions** |  |  |
| Cellular Development | 3.69E-02 - 2.44E-10 | 24 |
| Cellular Growth and Proliferation | 3.69E-02 - 2.44E-10 | 21 |
| Cellular Movement | 3.69E-02 - 5.18E-09 | 13 |
| Cell Death and Survival | 3.69E-02 - 6.48E-08 | 23 |
| Cell Cycle | 3.69E-02 - 1.93E-07 | 10 |
|  |  |  |
| **Physiological System Developnment and Function** |  |  |
| Organismal Functions | 2.59E-18 - 9.85E-19 | 11 |
| Digestive System Development and Function | 8.34E-17 - 8.34E-14 | 8 |
| Hepatic System Development and Function | 1.03E-03 - 8.34E-14 | 10 |
| Organ Development | 3.69E-02 - 8.34E-14 | 10 |

A summary of the top five diseases, cellular and physiological functions enriched by the 60 miRNA features. Note that the p-values are the lowest for the diseases category.

**Supplementary Table 2:** Cancer Types in the validation datasets.

**Supplementary Table 3:** Confusion matrix of the validation data set.

|  | EPIDERMIS | GI_TRACT | HEART | KIDNEY | LIVER | LUNG | LYMPH | NORMAL | PANCREAS | REPRODUCTIVE | STOMACH | THYROID | URINARY |
| --- | --- | --- | --- | --- | --- | --- | --- | --- | --- | --- | --- | --- | --- |
| EPIDERMIS | 6 | 0 | 0 | 0 | 0 | 0 | 0 | 0 | 0 | 0 | 0 | 0 | 0 |
| GI_TRACT | 0 | 7 | 0 | 1 | 0 | 0 | 1 | 2 | 1 | 0 | 0 | 0 | 0 |
| HEART | 0 | 0 | 0 | 0 | 0 | 0 | 2 | 0 | 0 | 0 | 0 | 0 | 0 |
| KIDNEY | 0 | 0 | 0 | 4 | 0 | 0 | 0 | 2 | 0 | 2 | 0 | 0 | 2 |
| LIVER | 0 | 2 | 0 | 0 | 0 | 0 | 0 | 0 | 1 | 0 | 0 | 0 | 0 |
| LUNG | 0 | 0 | 0 | 0 | 0 | 4 | 1 | 2 | 0 | 0 | 0 | 0 | 0 |
| LYMPH | 0 | 0 | 0 | 0 | 0 | 0 | 59 | 0 | 0 | 0 | 0 | 0 | 1 |
| NORMAL | 0 | 1 | 0 | 0 | 0 | 0 | 3 | 18 | 0 | 0 | 0 | 0 | 0 |
| PANCREAS | 0 | 0 | 0 | 0 | 0 | 0 | 0 | 0 | 7 | 0 | 0 | 0 | 0 |
| REPRODUCTIVE | 2 | 0 | 0 | 0 | 0 | 0 | 1 | 4 | 0 | 14 | 0 | 0 | 1 |
| STOMACH | 0 | 0 | 0 | 0 | 0 | 0 | 0 | 2 | 0 | 0 | 0 | 0 | 0 |
| THYROID | 0 | 0 | 0 | 0 | 0 | 2 | 10 | 0 | 0 | 0 | 0 | 0 | 0 |
| URINARY | 0 | 0 | 0 | 0 | 0 | 0 | 4 | 0 | 0 | 1 | 0 | 0 | 3 |

**Supplementary Table 4:** Sensitivity and Specificity of different cancer types in the validation dataset.

|  | **Sensitivity** | **Specificity** |
| --- | --- | --- |
| EPIDERMIS | 0.75 | 1.00 |
| GI_TRACT | 0.70 | 0.97 |
| HEART | - | 0.99 |
| KIDNEY | 0.80 | 0.96 |
| LIVER |  | 0.98 |
| LUNG | 0.67 | 0.98 |
| LYMPH | 0.73 | 0.99 |
| NORMAL | 0.60 | 0.97 |
| PANCREAS | 0.78 | 1.00 |
| REPRODUCTIVE | 0.82 | 0.95 |
| STOMACH | - | 0.99 |
| THYROID | - | 0.93 |
| URINARY | 0.43 | 0.97 |

The blank spaces for sensitivity are indicative of organ types that were not in our validation data, but were nevertheless predicted; e.g. there were no heart samples in our validation set, so the sensitivity is not calculable.
